# Sex disparity in skin carcinogenesis and potential influence of sex hormones

**DOI:** 10.1002/ski2.27

**Published:** 2021-04-01

**Authors:** V. Collier, M. Musicante, T. Patel, F. Liu‐Smith

**Affiliations:** ^1^ Kaplan‐Amonette Department of Dermatology The University of Tennessee Health Science Center Memphis Tennessee USA; ^2^ College of Medicine University of Tennessee Health Science Center Memphis Tennessee USA; ^3^ Department of Preventative Medicine University of Tennessee Health Science Center Memphis Tennessee USA

## Abstract

**Background:**

Sex or gender disparity in skin cancer has been documented for a long time at the population level. UV radiation (UVR) is a common environmental risk for all three major types of skin cancer: cutaneous melanoma (CM), basal cell carcinoma (BCC) and cutaneous squamous cell carcinoma (cSCC). The underlying mechanism for sex disparity has been largely attributed to sex‐differentiated behaviour patterns related to UVR. Non‐UVR factors such as intrinsic physiological differences have been suggested but remain understudied.

**Aims, Materials and Methods:**

This review summarizes and compares the known sex differences in three skin cancer types with regard to body site distribution and age influence.

**Results:**

We found a similar age‐dependent sex difference pattern in CM and BCC. Specifically, CM and BCC tend to show higher incidence in young women and old men, with a switching age around menopause. The switching age suggests involvement of sex hormones, which has shown controversial influence on skin cancers at epidemiological level. Literatures regarding sex hormone receptors for oestrogen, androgen and progesterone are summarized for potential explanations at molecular level.

**Discussion:**

Overall, more and more evidence suggests non‐UVR factors such as sex hormones play critical roles in skin cancer (especially CM and BCC), yet solid population and molecular evidence are required. Incidences of skin cancer are increasing which suggests limited effect for the current UVR‐avoidance prevention methods.

**Conclusion:**

Fully understanding the causes of sex disparities in incidence is necessary for developing a comprehensive prevention strategy.

## Graphical abstract

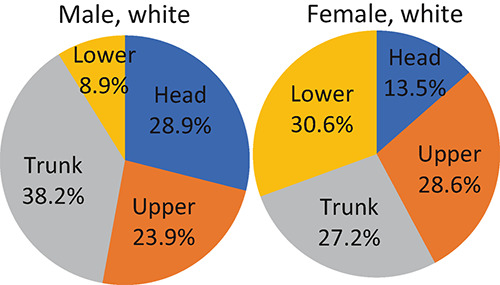

Sex difference is known in general for skin cancer and has been attributed to the difference of UVR‐related behavior. We found a similar pattern of sex difference in age‐dependent manner in CM and BCC, and summarized the possible links between sex hormones and sex disparity. These comparisons are critical for developing effective prevention strategies.

1

**What is already known about this topic?**

- Sex difference is known in general for skin cancer, and UVR impact is responsible.

**What does this study add?**

- Similar pattern of sex difference in age‐dependent manner in CM and BCC, and possible links between sex hormones skin cancer sex disparity. These comparisons are critical for developing effective prevention strategies.

## 2 INTRODUCTION

Sex difference has been documented and studied in various cancer types including skin cancer.^1^, ^2^, ^3^, ^4^ Molecular studies, in general, are lagging. Fully understanding sex differences at population and molecular levels will provide a basis for developing effective prevention strategies and treatment options for skin cancer.

As the outermost barrier organ in the human body, skin plays a unique role in defending the extrinsic physical and chemical insults such as UV radiation (UVR) and environmental carcinogens. Furthermore, skin is also capable of regulating responses to stress using a sophisticated skin neuroendocrine system.^5^, ^6^ Skin cancer includes three major types: cutaneous squamous cell carcinoma (cSCC), basal cell carcinoma (BCC) and cutaneous melanoma (CM). These cancers arise from different cell lineages including keratinocytes, basal cells, melanocytes and their precursor cells.^7^ Skin cancer incidences continue to increase in the past decades,^8^, ^9^ with UVR as a shared environmental risk factor. UVR includes solar and artificial such as sunbed use. Among the different wavelength of UV, UVC (100–280 nm) is mostly filtered by the atmosphere and thus is not directly received by human skin.^10^, ^11^ UVB (280–315 nm) is the major DNA mutagenic factor for cSCC, BCC and CM, while UVA (315–400 nm) also contributes to DNA mutations, likely through increased oxidative stress.^12^, ^13^

Sex‐differentiated exposure to UVR is related to behaviour differences in men and women, and at different ages. In fact, the behaviour difference was thought to be the main cause of the sex difference in skin cancer.^14^ Intrinsic risk factors such as sex hormones, on the other hand, are much less evaluated.^15^ Skin biology is profoundly influenced by sex hormones including oestrogen, testosterone and progesterone,^16^, ^17^, ^18^ which are primarily produced by sex organs but can be produced locally in skin tissue.^19^, ^20^ It has been elucidated that UVR impacts skin homeostasis through endocrine hormones including α‐melanocyte‐stimulating hormone, β‐endorphin, adrenocorticotropic hormone and serotonin, which can also be synthesized de novo in skin.^21^, ^22^, ^23^ Both sex hormones and endocrine hormones show regulatory effect on skin pigmentation,^24^, ^25^ which are tightly linked to skin carcinogenesis. Evidence of how sex hormones impact skin cancer has been emerging but requires further investigation.^26^ The intrinsic differences in these pathways in men and women may play crucial roles in explaining the observed sex difference in skin in addition to the behaviour difference, yet little solid conclusions have been reached thus far.^21^, ^27^, ^28^

CM has received much research attention because of its higher mortality rates. In contrast, BCC and cSCC (often grouped together as non‐melanoma skin cancer, NMSC) are less lethal and less studied, and are not reportable to cancer registries. NMSC counts for the most frequent malignancy in the United States and Canada and cost hundreds millions of dollars in care and treatment.^9^, ^29^ This review aims to summarize the current understanding of sex difference in CM, BCC and cSCC, with consideration of other variables including age and body sites. Sex hormones as potential underlying mechanisms of the sex differences will be reviewed and summarized.

## 3 CUTANEOUS MELANOMA

Overall, men are at higher risk than women for cutaneous melanoma.^30^ However, the sex difference changes follow the age axis: younger women and older men (cut‐off at ∼50 years of age) are at relatively higher risk than the opposite sex of the same age groups.^1^ UVR and the UVR‐related lifestyles are considered to be the cause of the observed sex difference; involvement of non‐UVR factors are consistently suggested but has been under‐explored.^31^, ^32^ Here we summarize the sex difference of CM incidence and the possible underlying causes.

**FIGURE 1:**
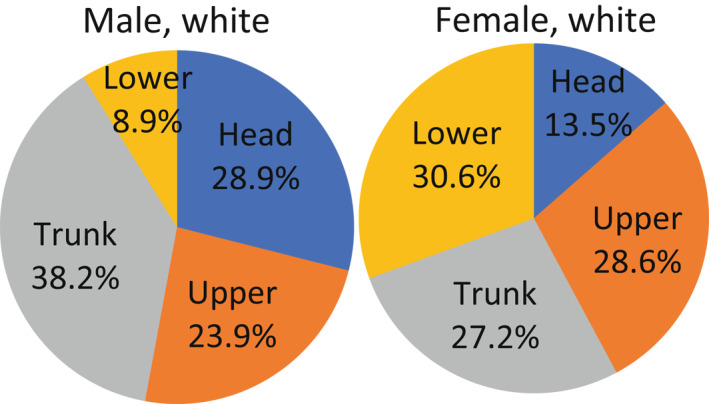
Body site distribution of cutaneous melanoma for white men and women. Data were obtained from US SEER 2000–2015. Head: head and neck; Upper: shoulder and upper extremities; Lower: hip and lower extremities

Men and women show distinct difference on the body sites. As shown in Figure 1, the majority of melanomas in men are found in trunk (38.2%), followed by head and neck (28.9%), shoulder and upper extremities (23.9%), and hip and lower extremities (8.9%). The majority of melanomas in women (30.6%) are in hip and lower extremities, followed by shoulder and upper extremities (28.6%), trunk (27.2%), and head and neck (13.5%) (Figure 1).^33^ The major contrasts are from hip and lower extremities where women showed extremely high incidence, and from head and neck where men showed higher incidence.^34^, ^35^, ^36^

Men and women also showed distinct difference in different age groups.^1^, ^37^ Younger women, defined as those less than 50 years of age, show a higher incidence than younger men. At age 50, the incidence rates of both sexes approach 1, indicating incidence is roughly equal between the genders. As age continues to increase, men surpass women, and the difference widens as age increases further. This relationship is demonstrated using SEER data from 2000 to 2015 as seen in Table 1.

**TABLE 1:** Age‐standardized cutaneous melanoma incidence rates and sex ratios, SEER 2000–2015

	Men		Women		
Age group	ASR	Std. err	ASR	Std. err	F/M
0–29	1.6	0.6	3.3	0.9	2.06
30–49	16.3	2.3	21.1	2.6	1.30
50–69	61.0	5.8	36.5	4.5	0.60
≥70	152.1	12.8	54.1	7.7	0.36
Overall	30.9	1.8	19.5	1.4	0.63

Abbreviations: ASR, age‐standardized rate; F/M, female/male.

As revealed in one of our previous publications, there is a distinct interaction between sex and UVI (UV radiation index).^38^ The age‐dependent sex difference in interaction with UVI is summarized in Table 2. According to a Poisson regression model,^38^ although UVI is consistently a promoting risk factor for melanoma at all ages, sex only interacts with UVI at older age (≥50 years) to influence CM risk. At younger age (<50 years), sex seems to independently impact CM risk, showing a promoting effect in females. Relative to males, females show a protection effect at older age (≥50 years).

**TABLE 2:** The sex and age impact on melanoma risk and their interactions with UVI

	Age	
Variables	<50	≥50
Female sex (relative to male)	Promotion	Protection
UVI	Promotion	Promotion
Statistical interaction between sex and UVI	No	Yes

Abbreviation: UVI, ultraviolet radiation index.

A study using county‐level data revealed that early‐stage melanomas diagnosed at older age (but not those diagnosed at younger age) were significantly associated with geographical average UVR for both men and women,^31^ further validating that melanomas diagnosed at a younger age may have less impact from UVR. A detailed analysis on melanoma gender difference in different races, age groups and anatomic sites also suggested differential mechanisms of early‐ and late‐onset melanoma.^34^ These observations argue against a sole role of UV or UV‐related behaviour in melanomas among the younger population. Interestingly, occupational outdoor work appears to not be a risk factor for CM.^39^ Rather, outdoor workers, most commonly male, who receive more cumulative UVR exhibit lower risk of CM than indoor workers who usually get intermittent sun exposure.^39^, ^40^ Therefore, UVR does not seem to impact CM in a dose‐dependent linear model.

Use of indoor tanning device complicates the age‐differentiated melanoma incidence outcome. Lazovich et al. observed that indoor tanning increases the odds for developing melanoma in young women but perhaps not in young men.^41^ Ever use of a tanning device increases melanoma risk,^42^ especially in women of age 45 years or younger (*P* < 0.05; odds ratio [OR] = 3.22).^43^ Fortunately, tanning device use has been declining since the International Agency for Research on Cancer (IARC) classified it as a group I carcinogen in 2009.^44^

The roles of intrinsic sex hormones in CM are less studied, perhaps due to controversy of epidemiological and molecular biological studies. First, it is generally accepted that pregnancy, oral contraceptive use or hormone replacement therapy (HRT) was not associated with melanoma risk,^40^, ^45^, ^46^, ^47^, ^48^, ^49^, ^50^ but controversies remain.^51^ A recent epidemiological study suggested that current menopausal HRT does significantly increase melanoma risk.^52^ This study adjusted for geographical UV exposure and was based on registry data that accurately recorded the hormone type and exposure duration. Additional compelling evidence came from a Swedish case–control study, in which high‐dose oestrogen (ethinylestradiol) was used to reduce growth in tall adolescent girls. The study found that odds for breast cancer were not different among treated and untreated girls, but odds for melanoma were extremely high in the treated group (OR = 6.1, *p* = 0.046).^53^

Oestrogen clearly affects the physiology of human skin and melanocytes but expression of oestrogen receptors (ERα and ERβ) in melanocytic lineage remains questionable.^54^, ^55^, ^56^, ^57^, ^58^, ^59^, ^60^ A recent publication was not able to detect ERα in cultured primary melanocytes even though 17‐β‐estradiol (E2) elicited a profound impact on MiTF expression and melanin synthesis.^25^ The authors found that a non‐canonical oestrogen receptor GPER1 played a crucial role.^25^ Another publication showed clear signal for ERα in human atypical nevi slides.^61^

Minimal studies have been carried out evaluating progesterone (P4) and its receptor, PGR (progesterone receptor). PR protein (encoded by PGR) in melanoma is controversial.^61^, ^62^ By using a number of different antibodies and positive control cell lines, we concluded that expression of PR in melanocytic lineage was not detectable at a protein level (Liu‐Smith Laboratory, unpublished data), which is consistent with our RNA‐Seq data and previously published data.^61^, ^63^ Perhaps other non‐canonical PRs support a function of P4 in melanoma, as recent epidemiological studies showed protective roles of progestin in melanoma in menopause hormone therapy (MHT).^52^, ^64^

Expression of AR was briefly reported before in melanocytes and nevi, only in melanocytes from genital area.^65^, ^66^ Recently, androgen receptor (AR), but not ERs, was found to be induced by acute UVB radiation in a fish melanoma model.^67^ The authors linked it to the differential sex difference in melanoma incidence in humans. Overall, studies regarding AR in melanoma are very limited.

## 4 BASAL CELL CARCINOMA

**FIGURE 2:**
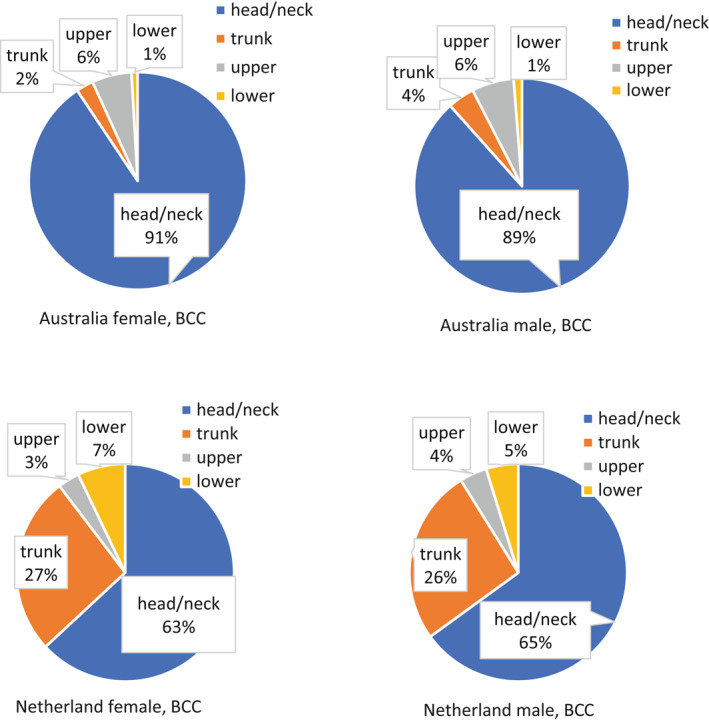
Body site distribution of basal cell carcinoma for white men and women. The pie chart was created based on data extracted from Buettner et al.^70^ (Australia), and Bastiaens et al.^71^ (The Netherlands), respectively. Head/neck: head and neck; Upper: shoulder and upper extremities; Lower: hip and lower extremities

BCC is the most common malignant tumour, with increasing incidence worldwide.^68^, ^69^ BCC most frequently appears on the head and neck.^68^, ^70^, ^71^ When the body sites are grouped into the same four groups described in Figure 1, there is no significant sex difference in distribution (Figure 2). Two previously published datasets, from Townsville, Australia, and from the Netherlands, are used to illustrate the distribution of BCCs.^70^, ^71^ The Australian cohort showed about 90% of their BCCs on the head and neck and a very small percent on the trunk (Figure 2a,b). The Netherlands group has only about 63%–65% of their BCCs on head and neck, with 26%–27% on their trunk. This body site distribution difference in different geographic regions may reflect the direct influence of solar UVR, as Australia has the highest annual average UVI on Earth (range 6.3–12.0), while the Netherlands annual average UVI was only 3.2.^38^ Additionally, indoor tanning may contribute to increased truncal BCCs in the Netherlands, as tanning is still viewed as fashionable in the Netherlands.^72^

Men tend to have a higher incidence on the ears, whereas women show a predilection for the lips and perioral region.^68^, ^70^, ^73^, ^74^, ^75^, ^76^, ^77^ These observations may be a result of differences in behaviour and lifestyle choices that are UVR‐associated. Men are more likely to have facial hair around the mouth, whereas women may be more likely to have hair styles that cover the ears. In either case, hair may serve a protective role against BCC.

Overall, men have a higher incidence rate of BCC than women, and with up to more than twice as likely than women to have greater than six NMSC tumours.^78^, ^79^ It has thus been suggested that male sex is an inherent risk factor for BCC development.^80^ Like CM, there is very similar age‐dependent sex difference in BCC incidence. BCC preferentially affects women at age 50 and younger.^68^, ^81^, ^82^ A meta‐study using 16 published BCC datasets revealed that the female incidence rates of BCC were higher than males at a young age, and the female/male (F/M) rate ratios approached 1.0 around age 50. After age 50, the male/female rate ratios increased with age.^80^ This age‐dependent sex difference of BCC is very similar to that of CM as outlined in Table 1. A few other studies also showed similar patterns. For example, we calculated the F/M incidence rate ratios from a study on BCC in Puerto Rico,^83^ which was 1.72, 0.84, 0.66 and 0.84 for age 20–34, 35–59, 60–84 and 85+, respectively. We also calculated the F/M incidence rate ratio to be 2.24, 1.19 and 0.71 for age groups of <40, 40–64 and ≥65 years from another study.^84^ A Mayo Clinic study using Olmsted County (Minnesota) data showed a similar age‐dependent sex difference in BCC, and the trend of age‐specific F/M incidence rate ratios (as a function of age) resembles that of cutaneous melanoma.^1^, ^68^

Incidence of BCC in young women (under 50) exhibited a fast increase than older women.^68^ A potential mechanism is perhaps tanning bed use among young women. It has been demonstrated that indoor tanning increases the risk of early onset BCC.^85^ However, other factors may also contribute to BCC increase in young women, such as smoking and history of blistering sunburns.^86^ Overall, the causes of the age‐dependent sex difference in BCC are largely unexplained.

A few studies have attempted to analyse the role of sex hormones in BCC by investigating associations with oral contraceptive pills (OCPs) and HRT. Kuklinski et al. demonstrated an elevated OR of BCC with long‐term OCP use.^87^ However, other studies have found no such association between BCC and OCP usage.^88^, ^89^ Data are similarly mixed in studies on BCC and HRT usage. Three individual studies have demonstrated a link between BCC and HRT; however, Tang et al. concluded that there is no association.^27^, ^88^, ^89^, ^90^ Notably, Tang et al. was limited to an approximately 6‐year follow‐up period, which may not have been enough time for BCC development.

On the molecular level, Rogers et al. demonstrated that BCCs lack oestrogen and progesterone receptors; however, a rat model study has shown that parenteral estradiol administration stimulates BCC onset and development.^91^, ^92^ These findings suggest that sex hormones may modulate BCC through an indirect mechanism. Mutations in the sonic hedgehog pathway transmembrane protein PTCH1 have long been understood to underlie many BCCs, and it has been shown that PTCH1 is involved in the response to steroids, such as oestrogen and progesterone.^93^, ^94^, ^95^ Heterozygous Ptch1^+/−^ mutant mice developed BCC after UVR in males but not in female mice.^96^ Ovariectomy of these Ptch1^+/−^ female mice restored their susceptibility to BCC induced by radiation or chemical carcinogens,^97^ suggesting involvement of female sex hormones.

## 5 CUTANEOUS SQUAMOUS CELL CARCINOMA

No SEER data reports cSCC; therefore, the annual new cases are only estimated with various models. Incidence of cSCC is estimated to range between 186 157 and 419 543 newly diagnosed cases in the United States in 2013,^98^ and 107.6 per 100 000 person‐years for men and 68.7 per 100 000 person‐years for women in the Netherlands in 2017.^99^ In a 2017 report, Muzic et al. found a 263% increase between 1976–1984 and 2000–2010 in a large‐scale study from Olmsted County, Minnesota.^68^ In the Netherlands, cSCC incidence increased ∼2.7‐fold in men and 4.9‐fold in women from 1989 to 2017.^99^

Although UVR is the predominant driving factor in both BCC and cSCC, different patterns are associated with BCC and cSCC: the intermittent intense episodes of UVR drives BCC tumorigenesis, while cSCC appears to be associated with life‐time cumulative solar UV exposure.^100^

While incidence rates of cSCC in men continue to be higher than that in women overall, the studies have shown that the rate of increase of incidence in women has surpassed that of men,^68^, ^101^, ^102^ thus the gap is decreasing. Additionally, comparing two reports tracking the incidence in Olmsted County, Minnesota, women and men under 40 years old had similar incidence rates of cSCC from 1976 to 2003^81^; women have surpassed men of the same age in overall incidence in 2000–2010 time period.^68^ These changes are interesting as they seem to bring the F/M rate ratios of cSCC (as a function of age) to resemble that of BCC and melanoma, that is, young women tend to show higher incidence than young men, while the rate ratio reverses at older age.

**FIGURE 3:**
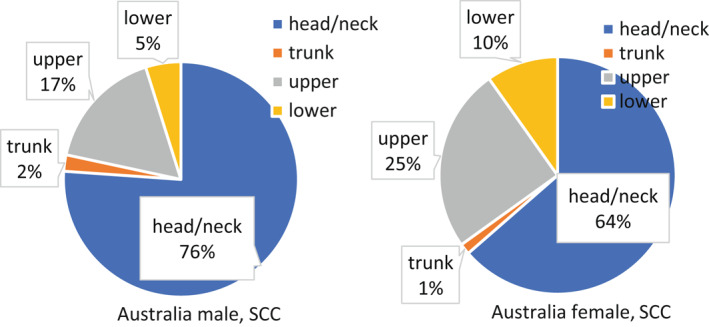
Body site distribution of cutaneous squamous cell carcinoma for men and women in Australia. The pie chart was created based on data extracted from Buettner et al.^70^ (Australia)

The face appears to be the most commonly affected body site in both sexes for cSCC.^68^, ^103^ However, men have a much higher incidence than women of tumours on the ears, scalp and neck, and trunk.^103^ As shown in Figure 3, based on data from Townsville, Australia,^70^ 76% of cSCC tumours occurred in head and neck region for men and 64% for women. Women have been found to have a slightly higher incidence of cSCC on the extremities^68^ (Figure 3). With regard to prognostic differences in men and women, men have higher rates of metastasis, and consequently, a higher likelihood of mortality.^104^

Higher incidence of cSCC in men is also attributed to differential sun exposure. Whether sex hormones play a role is unclear. A 2016 large‐scale case–control study showed a statically significant increased risk of SCC in users of OCPs, especially at doses above 50 mg of oestrogen. Menopausal HRT also significantly increased the SCC risk.^87^ Furthermore, when there was a combined history of OCP and HRT use, the OR of developing cSCC was higher than with either alone. Conversely, in a 2012 study from Denmark, OCP use was not found to be associated with cSCC at any duration, and menopausal HRT was only found to increase the risk of SCC with every 5 years of use.^89^ Tang et al.’s study showed no relationship between either of the keratinocyte cancers and MHT; however, this study was subject to a limited follow‐up period as discussed above.^27^

Little research has been carried out at molecular and cellular levels regarding the roles of sex hormones in cSCC. One study using a single cell line, A431, showed that oestrogen receptors ERα and G‐coupled protein receptor (GPR30, or GPER1) modulated expression of tumour markers Cyclin D1 and CD55; however, ERβ downregulated both tumour markers.^105^

## 6 DISCUSSION AND CONCLUSIONS

Notable commonalities among the three cutaneous malignancies are evidence of higher incidence in younger women than in younger men, overall lifetime increased incidence in men as compared to women, and UVR as one of the shared factors driving mutagenesis.^13^, ^61^, ^78^ When comparing the anatomical location for each tumour among the two genders, melanoma showed the greatest variation, while NMSCs showed minimal difference in location site between men and women. Although UVR is the common risk factor for skin cancer, it must be understood that UVR is also required for skin to synthesize vitamin D_3_, which shows a photoprotective effect against DNA damage in keratinocytes and melanocytes together with vitamin D_3_ metabolites.^106^ Additional studies showed vitamin D_3_ exhibited protective effect against cSCC, BCC and CM.^107^, ^108^, ^109^ There is no significant sex difference in serum vitamin D_3_ levels,^110^ but it is interesting to note that decreasing vitamin D3 levels are significantly associated with decreasing free testosterone levels in both men and women.^110^

Sex hormone influence on CM, BCC and cSCC remains controversial. Perhaps the most convincing evidence of sex hormone influence has been demonstrated for CM, but these results have been inconsistent as detailed above. The data available regarding MHT and OCP influence on NMSC are limited and contradictory. Molecular pathways involving sex hormones have been minimally evaluated in melanocytes and keratinocytes. As such, consistent expression of a known oestrogen receptor and downstream mechanisms of mutagenesis have yet to be fully elucidated.

Further exploration of the role of sex hormones in CM, BCC and cSCC would be of value to an effective prevention. Being able to correctly identify and modify risk factors for malignancy is of vital importance. Furthermore, understanding drivers of mutagenesis on a molecular level is key to the development of targeted therapies. UVR‐related behavioural choices have long been believed to account for the gender disparity in incidence. However, as tanning bed use decreases, yet incidence increases, and more light is shed on intrinsic risk factors, it has become apparent that the answer is likely multifactorial.^30^

## CONFLICT OF INTEREST

None of the authors has conflict of interest.
